# A novel nomogram for predicting non-infectious fever in patients following laparoscopic myomectomy

**DOI:** 10.1038/s41598-024-78666-y

**Published:** 2024-11-13

**Authors:** Yichen Zhu, Juntao Tan, Lin-Kang Liu, Buzhen Tan

**Affiliations:** 1 Department of Obstetrics & Gynecology, The Second Affiliated Hospital of Nanchang University, Nanchang, 330000 Jiangxi China; 2 Jiangxi Province Key Laboratory of Breast Diseases, The Third Hospital of Nanchang, Nanchang, 330008 Jiangxi China; 3 Department of Gynecology, PingXiang Maternal and Child Care, Pingxiang, 337000 Jiangxi China

**Keywords:** Laparoscopic myomectomy, Non-infectious fever, Nomogram, Predictive model, Health care, Medical research, Risk factors

## Abstract

This study aimed to develop and validate a novel nomogram to predict the risk of non-infectious fever (NIF) in patients following laparoscopic myomectomy. A retrospective analysis was conducted on data from patients who underwent laparoscopic myomectomy between 2019 and 2023. Pertinent variables before, during, and after surgery were collected. Multivariate logistic regression analysis identified independent risk factors for postoperative NIF, from which a nomogram was constructed. The study included 576 patients, among whom 64 (11.1%) developed postoperative NIF. Multivariate analysis identified leiomyoma size, number of leiomyomas, preoperative hemoglobin levels, operative time, and estimated blood loss as independent risk factors for postoperative NIF. A predictive nomogram model incorporating these factors demonstrated good accuracy following internal validation. The developed nomogram represents the first tool tailored for predicting NIF after laparoscopic myomectomy. Its implementation can assist clinicians in early identification of high-risk patients, facilitating timely preventive and management strategies.

## Introduction

Uterine fibroids, benign tumors originating from the uterine smooth muscle, are a common gynecologic condition^1^. Epidemiological data indicate that 20–40% of women of reproductive age develop uterine fibroids, with prevalence influenced by age, ethnicity, and lifestyle^2,3^. Studies have shown that while most uterine fibroids are asymptomatic, about 25% of women experience symptoms severe enough to require medical intervention^4,5^. Uterine fibroids present with diverse symptoms, including menorrhagia, irregular cycles, pelvic pain, infertility, and organ compression, all of which can significantly diminish quality of life^4,5^.

Previous research has established that laparoscopic myomectomy, compared to open surgery, offers several advantages such as reduced postoperative pain, shorter hospital stays, and quicker recovery^6,7^. However, despite these benefits, the procedure is not without risks. Complications, including non-infectious fever (NIF), have been identified, although their incidence and contributing factors remain underexplored.

Postoperative NIF is defined as a fever above 38.0 °C occurring within 48 h after surgery without evidence of infection^8,9^. Although NIF is usually self-limiting and not associated with long-term harm, it can cause patient discomfort, prolong hospitalization, increase costs, and sometimes obscure true infections, delaying treatment^10^. Thus, predicting and managing NIF in patients after laparoscopic myomectomy is essential for improving care and reducing costs.

To date, research on the incidence and predictors of NIF after laparoscopic uterine fibroid resection remains limited. While some studies have examined the general incidence of postoperative fever, detailed investigations into specific factors contributing to NIF are lacking^11,12^. Additionally, the mechanisms underlying NIF are not fully understood, complicating efforts to prevent or mitigate this complication. Therefore, developing an accurate predictive model based on potential risk factors is essential for early identification of high-risk patients, implementation of preventive measures, and optimization of postoperative care.

Nomograms are visual statistical models that provide personalized risk predictions by integrating multiple relevant factors^13^. Although widely used in oncology and cardiovascular fields, their application in predicting NIF after laparoscopic myomectomy is limited. Therefore, this study aims to develop and validate a novel nomogram model, incorporating clinical factors such as hemoglobin levels, leiomyoma size, operative time, and intraoperative blood loss, to provide a reliable tool for predicting NIF after laparoscopic myomectomy.

## Materials and methods

### Study design and setting

This study was conducted following the STROBE guidelines^14^, ensuring rigorous reporting of observational studies. Relevant aspects of study design, patient selection, data collection, statistical analysis, and interpretation were meticulously reported in accordance with these guidelines.

This retrospective cohort study enrolled female patients who underwent laparoscopic myomectomy at our hospital between June 2019 and May 2023. The inclusion criteria comprised: age between 18 and 50 years, a preoperative diagnosis of uterine fibroids confirmed by imaging studies (e.g., ultrasound or MRI), undergoing laparoscopic myomectomy as the surgical intervention, and availability of complete medical records, including preoperative, intraoperative, and postoperative data. The exclusion criteria included a history of acute or chronic infectious diseases, preoperative fever symptoms, concurrent major internal medical conditions such as cardiovascular diseases or malignancies, and the presence of immune system diseases or patients undergoing immunosuppressive therapy. To minimize selection bias, we employed a consecutive sampling method, enrolling all eligible patients who met the inclusion criteria during the specified study period. This approach ensured that the sample was representative of the population undergoing laparoscopic myomectomy at our institution. Additionally, a thorough review of medical records was conducted to confirm that all patients included in the study met the established criteria, thereby enhancing the validity of our findings.

## Data selection

Patient data were extracted from medical records, encompassing basic demographic information (age, weight, height, BMI, etc.), medical history (size and number of fibroids, etc.), preoperative laboratory results (hemoglobin and albumin levels), and surgical details (duration of surgery, intraoperative blood loss, endometrial penetration status, etc.). The primary outcome of this study was the incidence of postoperative NIF, defined as a temperature ≥ 38 °C occurring within 48 h after surgery without any infectious cause. Secondary endpoints included operative time, estimated blood loss, length of hospital stay, and changes in preoperative hemoglobin levels. Data were systematically collected to evaluate these outcomes, with a focus on identifying independent predictive factors associated with NIF. The endpoints were chosen based on their clinical relevance and potential impact on postoperative recovery and patient management.

## Definition of postoperative NIF

Postoperative NIF was defined as a body temperature of ≥ 38.0 °C persisting for three days or more following surgery, subsequent to a comprehensive clinical and laboratory evaluation ruling out sources of infection such as postoperative wound infections or urinary tract infections^8,10^.

## Intervention/Treatment description

In this study, all patients underwent laparoscopic myomectomy, a minimally invasive surgical procedure aimed at removing uterine fibroids while preserving the uterus. The intervention was performed under general anesthesia, and standard laparoscopic techniques were employed. Initially, three to four small incisions (typically 5–10 mm) were made in the abdominal wall to insert the laparoscope and surgical instruments. Carbon dioxide was used to inflate the abdominal cavity, providing better visualization of the pelvic organs. Once access was achieved, the surgeon identified the fibroids using the laparoscope. Depending on the size and location of the fibroids, various techniques were utilized for their removal, including enucleation and morcellation. Enucleation involved carefully dissecting the fibroid from the surrounding uterine tissue, while morcellation was used for larger fibroids to facilitate their removal through the small incisions. After the fibroids were excised, the uterine wall was sutured closed using absorbable sutures. Postoperatively, patients were monitored in the recovery area for complications such as bleeding or infection.

## Statistical analyses

All statistical analyses were performed using SPSS version 26.0 and R software version 4.1.1. Patients were randomly allocated into training and validation groups at a ratio of 7:3 using R software to ensure the model’s robustness and discriminative ability. Descriptive statistics summarized all variables, with continuous variables presented as mean ± standard deviation (SD) and categorical variables as frequency (percentage). Univariate analyses, including chi-square tests and t-tests, were conducted to identify potential risk factors associated with postoperative NIF. Variables demonstrating significance (P < 0.05) were entered into multivariate logistic regression analysis to determine independent predictors of postoperative NIF. Based on the results of multivariate analysis, a nomogram prediction model was constructed using the ‘rms’ package in R software. Harrell’s concordance index (C-index) and the area under the curve (AUC) were utilized to evaluate the nomogram’s predictive accuracy and discriminative ability. Calibration curves generated using the ‘calibration curve’ package assessed the consistency between predicted and observed outcomes. Model robustness was verified through 1000 bootstrapping resamples. Decision curve analysis (DCA) was performed using the ‘rmda’ package to evaluate the clinical utility and net benefit of the nomogram.

### Ethics statement

The study was approved by the ethics committee of The Second Affiliated Hospital of Nanchang University, adhering to the principles of the Helsinki Declaration. Written informed consent was obtained from all participants in accordance with Helsinki Declaration guidelines.

## Results

### Clinicopathologic characteristics

**Fig. 1:**
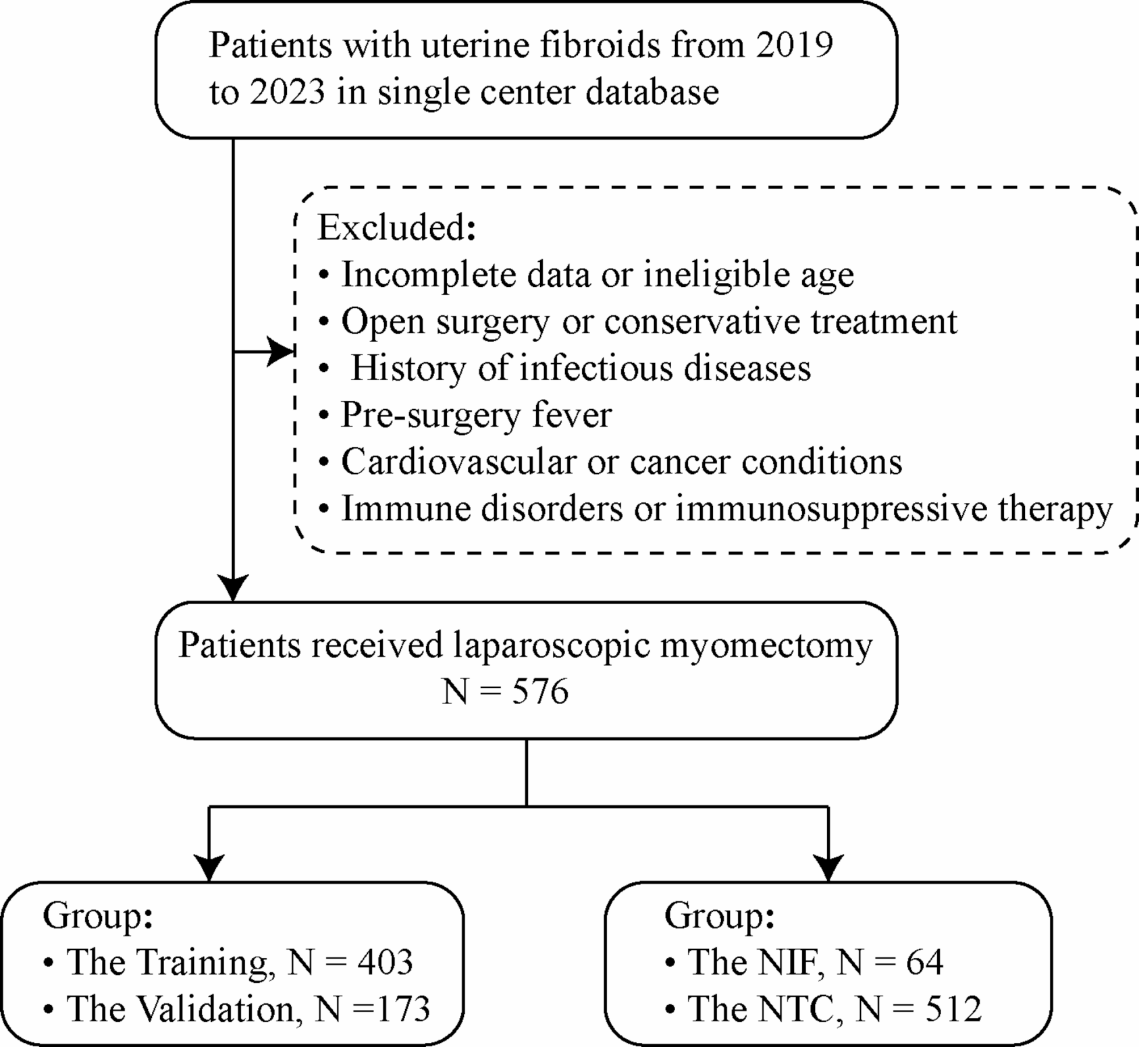
Flow chat of selecting patients in the study. NIF Non-infectious fever, NTC normal temperature control.

From 2019 to 2023, patients with uterine fibroids were screened from a single-center database for inclusion in this study. After applying inclusion and exclusion criteria, a total of 576 patients were enrolled (Fig. 1). Using R software, these patients were randomly assigned to training (419 cases) and validation groups (176 cases) at a 7:3 ratio. There were no significant differences in clinical characteristics between the training and validation groups (*P* > 0.05) (Table 1).

**Table 1:** Patient characteristics for the 576 study participants.

Variables	Total (*n* = 576)	Training set (*n* = 403)	Validation set (*n* = 173)	*P*	NIF (*n* = 64)	NTC (*n* = 512)	*P*
Age (yrs), n (%) < 40 ≥ 40	141 (24.5) 435 (75.5)	104 (25.8) 299 (71.2)	37 (21.4) 136 (78.6)	0.258	14 (21.9) 50 (78.1)	127 (24.8) 385 (75.2)	0.607
BMI (kg/m^2^) < 25 ≥ 25	395 (68.6) 181 (31.4)	282 (70.0) 121 (30.0)	113 (65.3) 60 (34.7)	0.270	46 (71.9) 18 (28.1)	349 (68.2) 163 (31.8)	0.547
Hypertension, n (%) No Yes	524 (91.0) 52 (9.0)	366 (90.8) 37 (9.2)	158 (91.3) 15 (8.7)	0.845	59 (92.2) 5 (7.8)	465 (90.8) 47 (9.2)	0.719
Diabetes, n (%) No Yes	536 (93.1) 40 (6.9)	379 (94.0) 24 (6.0)	157 (90.7) 16 (9.3)	0.154	60 (93.8) 4 (6.3)	476 (93.0) 36 (7.0)	0.817
Hyperlipidemia, n (%) No Yes	501 (87.0) 75 (13.0)	345 (85.6) 58 (14.4)	156 (90.2) 17 (9.8)	0.136	57 (89.1) 7 (10.9)	444 (86.7) 68 (13.3)	0.599
History of abdominal surgery, n (%) No Yes	403 (69.9) 173 (30.1)	286 (71.0) 117 (29.0)	117 (67.6) 56 (32.4)	0.423	42 (65.6) 22 (34.4)	361 (70.5) 151 (29.5)	0.422
Location of leiomyoma, n (%) Submucosal Intramural Subserosal	40 (6.9) 285 (49.5) 251 (43.6)	23 (5.7) 207 (51.4) 173 (42.9)	17 (9.8) 78 (45.1) 78 (45.1)	0.132	5 (7.8) 31 (48.4) 28 (43.8)	35 (6.8) 254 (49.6) 223 (43.6)	0.954
Size of leiomyoma (cm), n (%) < 6 ≥ 6	397 (68.9) 179 (31.1)	281 (69.7) 122 (30.3)	116 (67.1) 57 (32.9)	0.525	31 (48.4) 33 (51.6)	366 (71.5) 146 (28.5)	< 0.001
Number of leiomyomas, n (%) < 3 ≥ 3	301 (52.3) 275 (47.7)	216 (53.6) 187 (46.4)	85 (49.1) 88 (50.9)	0.325	25 (39.1) 39 (60.9)	276 (53.9) 236 (46.1)	0.025
Preoperative albumin (g/L), n (%) < 35 ≥ 35	127 (22.0) 449 (78.0)	94 (23.3) 309 (76.7)	33 (19.1) 140 (80.9)	0.259	20 (31.3) 44 (68.8)	107 (20.9) 405 (79.1)	0.060
Preoperative hemoglobin (g/L), n (%) < 110 ≥ 110	146 (25.3) 430 (74.7)	99 (24.6) 304 (75.4)	47 (27.2) 126 (72.8)	0.511	25 (39.1) 39 (60.9)	121 (23.6) 391 (76.4)	0.007
Perioperative antibiotic usage, n (%) No Yes	143 (24.8) 433 (75.2)	103 (25.6) 300 (74.4)	40 (23.1) 133 (76.9)	0.535	17 (26.6) 47 (73.4)	126 (24.6) 386 (75.4)	0.733
Operative time (hours), n (%) < 2 ≥ 2	404 (70.1) 172 (29.9)	284 (70.5) 119 (29.5)	120 (69.4) 53 (30.6)	0.790	37 (57.8) 27 (42.2)	367 (71.7) 145 (28.3)	0.022
Estimate blood loss (ml), n (%) < 200 ≥ 200	440 (76.4) 136 (23.6)	313 (77.7) 90 (22.3)	127 (73.4) 46 (26.6)	0.270	40 (62.5) 24 (37.5)	395 (77.1) 117 (22.9)	0.006
Endometrial penetration status, n (%) No Yes	484 (84.0) 92 (16.0)	334 (82.9) 69 (17.1)	140 (80.9) 33 (19.1)	0.573	53 (82.8) 11 (17.2)	431 (84.2) 81 (15.8)	0.778
Volume of drainage (ml)	95.45 ± 22.96	96.15 ± 23.39	94.72 ± 21.87	0.493	97.87 ± 24.69	94.55 ± 23.72	0.294
Drainage indwelling time (d)	1.35 ± 0.47	1.33 ± 0.51	1.37 ± 0.49	0.383	1.33 ± 0.50	1.38 ± 0.50	0.451
Length of hospital of stay (d), n (%) < 7 ≥ 7	496 (86.1) 80 (13.9)	350 (86.8) 53 (13.2)	146 (84.4) 27 (15.6)		54 (84.4) 10 (15.6)	442 (86.3) 70 (13.7)	0.670

NIF non-infectious fever, NTC normal temperature control, BMI body mass index.

Furthermore, patients were categorized into the NIF group (64 cases) and the normal temperature control (NTC) group (512 cases). The postoperative incidence rate of NIF was 11.1%. Age distribution, BMI, hypertension, diabetes, hyperlipidemia, history of abdominal surgery, location of leiomyoma, preoperative albumin levels, perioperative antibiotic usage, endometrial penetration status, volume of drainage, drainage indwelling time, and length of hospital stay did not exhibit significant differences between the NIF and NTC groups (all *P* > 0.05). However, notable differences were observed in the size of leiomyoma, number of leiomyomas, preoperative hemoglobin levels, operative time, and estimated blood loss. The NIF group had a higher proportion of patients with leiomyomas ≥ 6 cm (51.6% vs. 28.5%, *P* < 0.001), a higher percentage of patients with ≥ 3 leiomyomas (60.9% vs. 46.1%, *P* = 0.025), lower preoperative hemoglobin levels (39.1% < 110 g/L vs. 23.6%, *p* = 0.007), longer operative times (42.2% ≥ 2 h vs. 28.3%, *P* = 0.022), and greater estimated blood loss (37.5% ≥ 200 ml vs. 22.9%, *P* = 0.006) compared to the NTC group.

### Independent predictive factors in patients with NIF

Univariate analysis revealed five variables significantly associated with postoperative NIF: size of leiomyoma, number of leiomyomas, preoperative hemoglobin levels, operative time, and estimated blood loss (all *P* < 0.05). Subsequent multivariate logistic regression analysis confirmed these five variables—size of leiomyoma, number of leiomyomas, preoperative hemoglobin levels, operative time, and estimated blood loss—as independent predictive factors (Table 2).

**Table 2:** Logistic regression analysis of the predictors for the risk of postoperative NIF.

Variables	OR (95%CI)	*P*
Size of leiomyoma (cm) < 6 ≥ 6	1.00 (Reference) 2.67 (1.58–4.52)	< 0.001
Number of leiomyomas < 3 ≥ 3	1.00 (Reference) 1.82 (1.07–3.10)	0.027
Preoperative hemoglobin (g/L) < 110 ≥ 110	1.00 (Reference) 0.48 (0.28–0.82)	0.008
Operative time (hours) < 2 ≥ 2	1.00 (Reference) 0.54 (0.32–0.92)	0.024
Estimate blood loss (ml) < 200 ≥ 200	1.00 (Reference) 0.49 (0.29–0.85)	0.011

NIF Non-infectious fever.

### Development and validation of the Nomogram

**Fig. 2:**
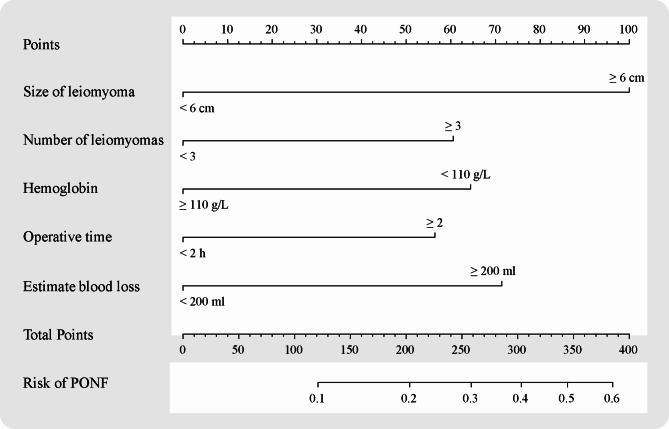
The graph showed nomogram for predicting PONF.

Based on the identified independent predictive factors, we developed a nomogram to predict NIF following laparoscopic myomectomy. Figure 2 illustrates the nomogram, incorporating five variables: size of leiomyoma, number of leiomyomas, preoperative hemoglobin levels, operative time, and estimated blood loss. Each variable is assigned different scores: size of leiomyoma < 6 cm (0 points), ≥ 6 cm (100 points); number of leiomyomas: < 3 (0 points), ≥ 6 (61.25 points); preoperative hemoglobin levels: < 110 g/L (63.75 points), ≥ 110 g/L (0 points); operative time: < 2 h (0 points), ≥ 2 h (55 points); estimated blood loss: < 200 ml (0 points), ≥ 200 ml (71.25 points). The total score is obtained by summing these five components, enabling prediction of NIF risk following laparoscopic myomectomy and facilitating clinical decision-making.

**Fig. 3:**
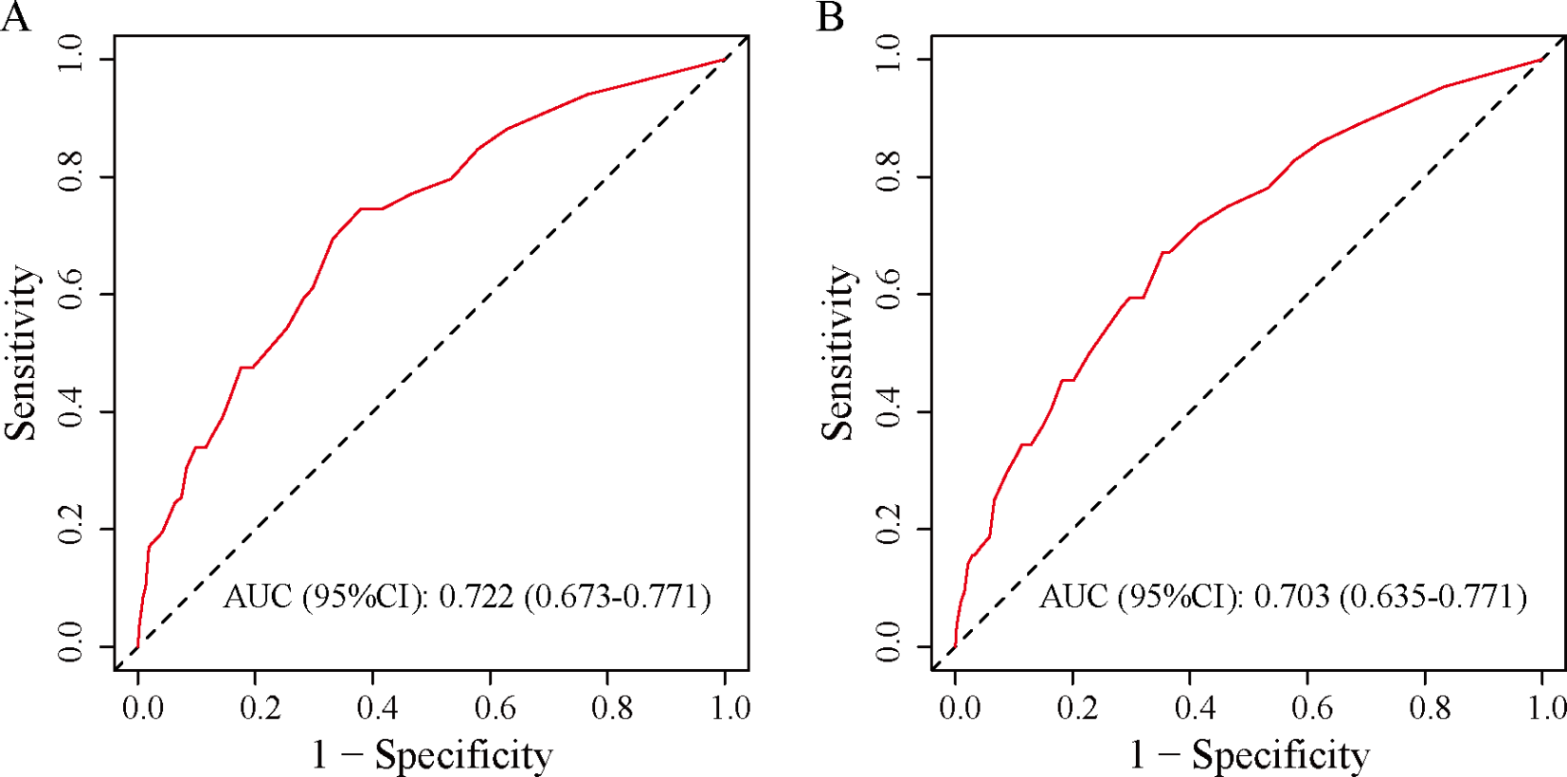
Model Validation Using ROC Curves in the Training (**A**) and Validation (**B**) Sets. ROC receiver operating characteristic curve.

**Fig. 4:**
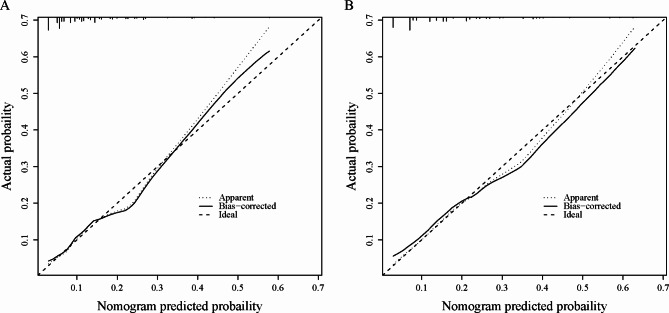
Model validation of calibration plot in the Training (**A**) and Validation (**B**) Sets. DCA decision curve analysis.

**Fig. 5:**
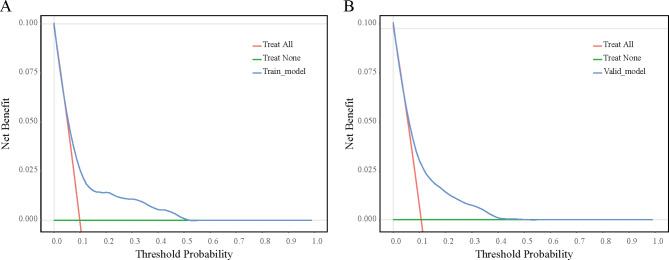
Model validation of DCA in the Training (**A**) and Validation (**B**) Sets. DCA decision curve analysis.

The nomogram’s overall performance was evaluated, with the training set achieving a C-index of 0.722 and the validation set yielding a C-index of 0.703, indicating robust discriminative capability of the predictive model. Additionally, ROC curve analysis revealed an AUC of 0.722 (95% CI: 0.673–0.771) for the training group and 0.703 (95% CI: 0.635–0.771) for the validation group (Fig. 3), confirming the model’s effectiveness in distinguishing outcomes. Furthermore, calibration curves for both the training and validation sets demonstrated high consistency between the nomogram-predicted probabilities and observed outcomes (Fig. 4). Decision curve analysis illustrated threshold probabilities for the predictive model ranging from 8 to 52% in the training set and from 8 to 41% in the validation set (Fig. 5), indicating its clinical utility across various risk thresholds.

## Discussion

This study identified key independent risk factors for the occurrence of NIF following laparoscopic myomectomy, including the size and number of leiomyomas, preoperative hemoglobin levels, operative time, and estimated blood loss. Using these variables, we developed and validated a predictive nomogram that demonstrated good accuracy in both the training and validation sets. The nomogram’s performance, assessed by C-index and AUC values, indicated a strong predictive capability, with calibration curves and decision curve analysis supporting its clinical utility.

Postoperative NIF is a common occurrence following laparoscopic procedures, particularly in gynecological surgeries^15,16^. While often transient and benign, NIF can lead to unnecessary antibiotic use, increased medical costs, and prolonged hospital stays, emphasizing the clinical relevance of predicting NIF in potentially affected patients for timely management and intervention.

The size of leiomyoma emerged as a crucial predictor for postoperative NIF, with larger leiomyomas (≥ 6 cm) associated with higher fever risks^17^. This finding is consistent with previous research, suggesting that larger leiomyomas may increase intraoperative trauma, leading to more pronounced systemic inflammatory responses and postoperative complications^18,19^. For example, a study by Hinchcliff and Cohen found that larger myomas are associated with increased surgical complexity, which correlates with higher rates of postoperative fever^20^. Moreover, the number of leiomyomas was identified as an independent predictor, with patients having three or more leiomyomas at higher risk of NIF. This suggests that the presence of multiple leiomyomas may complicate the surgical process and increase the likelihood of postoperative fever. This may be attributed to larger surgical wounds, increased postoperative hematoma absorption, and the effect of multiple leiomyomas on uterine contraction, leading to intraoperative bleeding and postoperative wound oozing, while pelvic blood absorption may also cause fever^19,21,22^. Similarly, Yorita et al. reported that the presence of multiple leiomyomas significantly increases the risk of complications, including fever, due to the extensive surgical dissection required^23^.

A low preoperative hemoglobin level has been identified as an independent predictor of postoperative NIF risk in patients undergoing laparoscopic myomectomy. This association may reflect poorer preoperative nutritional status or underlying chronic illness in patients, factors that not only may affect patients’ overall recovery capacity but also their response to surgical stress. Specifically, lower hemoglobin levels (< 110 g/L) were associated with higher fever incidence rates^24^. This scenario may occur because anemia can impair immune function and increase susceptibility to postoperative complications, including fever. Anemic patients may have lower tolerance to postoperative inflammatory responses^25^, making fever symptoms more likely^26^. This finding aligns with the work of Chen et al., who found that anemic patients undergoing gynecological surgery had a higher incidence of postoperative complications, including NIF, due to impaired immune function and reduced oxygen-carrying capacity^27^. Therefore, optimizing hemoglobin levels through iron supplementation or blood transfusion preoperatively may not only improve patient nutrition and mitigate the negative effects of chronic diseases but also help reduce the risk of postoperative NIF.

Prolonged operative time and increased estimated blood loss were identified as additional independent predictors of postoperative NIF. These factors not only reflect the complexity of surgery, intraoperative trauma, and the extent of inflammatory response but also closely relate to surgical trauma and tissue damage. Prolonged operative time may not only lead to excessive activation of the immune system but also trigger intense inflammatory responses^28,29^. Opsomer et al. similarly highlighted that longer operative times increase the likelihood of postoperative fever due to prolonged tissue exposure and the resulting inflammatory response^30^. Additionally, significant blood loss during surgery may lead to tissue hypoxia, further activating inflammatory pathways and ultimately causing fever^31,32^. These findings underscore the importance of measures to reduce operative time and control bleeding volume during laparoscopic myomectomy. Adopting optimized surgical techniques, meticulous hemostatic measures, and careful tissue handling strategies can not only mitigate intraoperative trauma and inflammatory responses but also effectively reduce the risk of postoperative fever.

Other clinical factors such as age distribution, BMI, hypertension, diabetes, hyperlipidemia, history of abdominal surgery, location of leiomyoma, preoperative albumin, perioperative antibiotic usage, endometrial penetration status, volume of drainage, drainage indwelling time, and length of hospital stay were not found to be associated with postoperative NIF. It is worth noting that although the association between preoperative albumin levels below 35 g/L and postoperative fever did not reach statistical significance (*P*= 0.06), the trend still suggests a possible correlation between low albumin and postoperative fever. Low albumin levels typically indicate malnutrition and inflammation, which may weaken the immune system and increase the risk of infection and postoperative fever^33–35^. Although there is no definitive causal evidence, given the close relationship between albumin levels and health status, its potential impact deserves attention. Therefore, it is recommended that physicians pay attention to patients’ nutrition and albumin levels preoperatively, and reduce the possibility of postoperative fever through nutritional interventions.

Given the significant associations identified between NIF and factors such as leiomyoma size, number of leiomyomas, preoperative hemoglobin levels, operative time, and estimated blood loss, future research should focus on developing targeted interventions to mitigate NIF risk. Studies could explore the impact of preoperative optimization of hemoglobin levels and the implementation of intraoperative strategies designed to minimize blood loss and reduce operative time on the incidence of NIF. Additionally, comparing the effectiveness of different surgical techniques in reducing NIF risk across varying leiomyoma sizes and numbers could provide valuable insights. Multi-center trials would further help to validate and refine the predictive nomogram across diverse patient populations, enhancing its clinical applicability.

The predictive model we developed, which demonstrated strong accuracy through internal validation, offers significant potential for clinical application. By assessing the risk of NIF preoperatively, clinicians can implement preventive measures such as optimizing preoperative preparation, improving patient nutrition, and planning surgical strategies to reduce operative time and blood loss. Moreover, this model can also guide postoperative management by enabling clinicians to monitor and intervene more effectively with high-risk patients, ultimately improving patient outcomes.

However, this study also has some limitations. Firstly, as a retrospective study, its data are derived from a single center, which may introduce selection bias and information bias. Secondly, although our model demonstrated good robustness in internal validation, external validation in other centers and larger patient populations is still needed to further confirm its universality and accuracy. Additionally, our study focused solely on non-infectious fever, failing to cover all possible postoperative complications. Future research could consider more postoperative scenarios to provide a more comprehensive predictive model.

Despite these limitations, our study provides valuable insights into the prediction and management of postoperative NIF in patients undergoing laparoscopic myomectomy. By identifying independent risk factors and developing a predictive model, we offer clinicians a tool to assess NIF risk more accurately and guide timely interventions. Future research endeavors should focus on further refining and validating the model across diverse patient populations to enhance its clinical utility.

## Conclusion

In summary, our study has developed and validated a nomogram for predicting postoperative NIF in laparoscopic myomectomy patients. This nomogram integrates key risk factors to provide clinicians with precise risk assessments and treatment recommendations. It can aid clinicians in early identification of high-risk patients, thereby enabling appropriate preventive and management measures to be taken. We anticipate its broad application in clinical practice and further validation across diverse patient populations to enhance its clinical utility.

## Data Availability

The primary data can be acquired from the corresponding authors in compliance with privacy and ethical constraints.

## References

[1] Stewart, E. A. et al. Uterine fibroids. *Nat. Rev. Dis. Primers*. **2**, 16043. 10.1038/nrdp.2016.43 (2016). 27335259 10.1038/nrdp.2016.43

[2] Ferrero, S., Vellone, V. G. & Barra, F. Pharmacokinetic drug evaluation of ulipristal acetate for the treatment of uterine fibroids. *Expert Opin. Drug Metab. Toxicol.* **14**, 107–116. 10.1080/17425255.2018.1417389 (2018). 29250979 10.1080/17425255.2018.1417389

[3] Stewart, E. A., Cookson, C. L., Gandolfo, R. A. & Schulze-Rath, R. Epidemiology of uterine fibroids: a systematic review. *Bjog*. **124**, 1501–1512. 10.1111/1471-0528.14640 (2017). 28296146 10.1111/1471-0528.14640

[4] Go, V. A. A. et al. A systematic review of the psychosocial impact of fibroids before and after treatment. *Am. J. Obstet. Gynecol.* **223**, 674–708e678. 10.1016/j.ajog.2020.05.044 (2020). 32474012 10.1016/j.ajog.2020.05.044

[5] Lin, L. C. et al. Oxidative stress mediates the inhibitory effects of Manzamine A on uterine leiomyoma cell proliferation and extracellular matrix deposition via SOAT inhibition. *Redox Biol.* **66**, 102861. 10.1016/j.redox.2023.102861 (2023). 37666118 10.1016/j.redox.2023.102861 PMC10491796

[6] Schneyer, R. J. et al. Development and validation of a simulation model for laparoscopic myomectomy. *Am. J. Obstet. Gynecol.* **227** 304.e301-304.e309 (2022). 10.1016/j.ajog.2022.04.042 35489440

[7] Leonardi, M., Robledo, K. P., Gordijn, S. J. & Condous, G. A consensus-based core feature set for surgical complexity at laparoscopic hysterectomy. *Am. J. Obstet. Gynecol.* **226**, 700. .e701-700.e709 (2022). 10.1016/j.ajog.2021.10.042 34785175

[8] Garibaldi, R. A., Brodine, S., Matsumiya, S. & Coleman, M. Evidence for the non-infectious etiology of early postoperative fever. *Infect. Control*. **6**, 273–277. 10.1017/s0195941700061749 (1985). 3847403 10.1017/s0195941700061749

[9] Xu, N. et al. Fluid homeostasis may predict the prognosis of non-infectious fever after total knee arthroplasty within 7-Day: a retrospective cohort study. *Front. Surg.* **8**, 690803. 10.3389/fsurg.2021.690803 (2021). 34604292 10.3389/fsurg.2021.690803 PMC8484697

[10] Maday, K. R., Hurt, J. B., Harrelson, P. & Porterfield, J. Evaluating postoperative fever. *Jaapa*. **29**, 23–28. 10.1097/01.JAA.0000496951.72463.de (2016). 27623291 10.1097/01.JAA.0000496951.72463.de

[11] Shim, Y. E. et al. Serum procalcitonin as a biomarker for differentiating between infectious and non-infectious fever after pancreas transplantation. *Clin. Transpl.* **35**, e14224. 10.1111/ctr.14224 (2021). 10.1111/ctr.14224 33438286

[12] Lu, Z. H., Lin, T. Y., Huang, H. S. & Liu, C. J. Preoperative urine analysis is An Effective Tool to Predict Fever after Miniaturized Percutaneous Nephrolithotomy on large Renal stones. *Urol. J.* **18**, 600–607. 10.22037/uj.v18i.6463 (2021). 34549802 10.22037/uj.v18i.6463

[13] Hu, H., Hu, L., Deng, Z. & Jiang, Q. A prognostic nomogram for recurrence survival in post-surgical patients with varicose veins of the lower extremities. *Sci. Rep.* **14**, 5486. 10.1038/s41598-024-55812-0 (2024). 38448552 10.1038/s41598-024-55812-0 PMC10918178

[14] von Elm, E. et al. Strengthening the reporting of Observational studies in Epidemiology (STROBE) statement: guidelines for reporting observational studies. *Bmj*. **335**, 806–808. 10.1136/bmj.39335.541782.AD (2007). 17947786 10.1136/bmj.39335.541782.AD PMC2034723

[15] Falagas, M. E. & Kasiakou, S. K. Mesh-related infections after hernia repair surgery. *Clin. Microbiol. Infect.* **11**, 3–8. 10.1111/j.1469-0691.2004.01014.x (2005). 15649297 10.1111/j.1469-0691.2004.01014.x

[16] Shen, W. & Lai, S. Diagnostic value of sCD163 combined with PCT and HS-CRP in patients with gynecological malignant tumors and fever. *J. Coll. Physicians Surg. Pak*. **30**, 1053–1057. 10.29271/jcpsp.2020.10.1053 (2020). 33143826 10.29271/jcpsp.2020.10.1053

[17] Huang, S. Y. et al. The impact of a novel transendometrial approach for caesarean myomectomy on obstetric outcomes of subsequent pregnancy: a longitudinal panel study. *Bjog*. **125**, 495–500. 10.1111/1471-0528.14798 (2018). 28646578 10.1111/1471-0528.14798

[18] Sheu, B. C., Huang, K. J., Huang, S. C. & Chang, W. C. Comparison of uterine scarring between robot-assisted laparoscopic myomectomy and conventional laparoscopic myomectomy. *J. Obstet. Gynaecol.* **40**, 974–980. 10.1080/01443615.2019.1678015 (2020). 31790613 10.1080/01443615.2019.1678015

[19] Kim, M. J., Lee, K., Park, J. Y., Jo, J. H. & Park, I. Y. The trend in cesarean myomectomies and the risk of obstetrical complications in Korea. *BMC Pregnancy Childbirth*. **22**, 387. 10.1186/s12884-022-04674-3 (2022). 35505300 10.1186/s12884-022-04674-3 PMC9066846

[20] Hinchcliff, E. M. & Cohen, S. L. Laparoscopic hysterectomy for uterine fibroids: is it safe? *Clin. Obstet. Gynecol.* **59**, 66–72. 10.1097/grf.0000000000000165 (2016). 26670837 10.1097/GRF.0000000000000165

[21] Nezhat, C., Nezhat, F., Silfen, S. L., Schaffer, N. & Evans, D. Laparoscopic myomectomy. *Int. J. Fertil.* **36**, 275–280 (1991). 1683655

[22] Peker, N., Gündoğan, S. & Şendağ, F. Laparoscopic management of huge Myoma Nascendi. *J. Minim. Invasive Gynecol.* **24**, 347–348. 10.1016/j.jmig.2016.09.003 (2017). 27632930 10.1016/j.jmig.2016.09.003

[23] Yorita, K., Nakagawa, T., Hirano, K. & Nakatani, K. Schwannoma-like uterine leiomyoma with fever of unknown origin and surgical management in a middle-aged woman: a case report. *Radiol. Case Rep.* **18**, 1691–1694. 10.1016/j.radcr.2023.01.094 (2023). 36895891 10.1016/j.radcr.2023.01.094 PMC9989318

[24] Kuo, H. C., Liu, S. F., Lin, P. X., Yang, K. D. & Lin, B. S. Near Infrared Spectroscopy Detects Change of Tissue Hemoglobin and Water Levelsin Kawasaki Disease and Coronary Artery lesions. *Child. (Basel)*. **9**10.3390/children9030299 (2022). 10.3390/children9030299 PMC8947440 35327671

[25] Saad, R. A. & Qutob, H. M. The relationship between anemia and obesity. *Expert Rev. Hematol.* **15**, 911–926. 10.1080/17474086.2022.2131521 (2022). 36189499 10.1080/17474086.2022.2131521

[26] Wen, A. et al. Exploration of the risk factors of Anemia in patients with tuberculous meningitis in South China. *Neuropsychiatr Dis. Treat.* **19**, 369–377. 10.2147/ndt.S391751 (2023). 36814696 10.2147/NDT.S391751 PMC9940599

[27] Chen, C. et al. Association of anemia and COVID-19 in hospitalized patients. *Future Virol.*10.2217/fvl-2021-0044 (2021). 34290821 10.2217/fvl-2021-0044 PMC8270514

[28] Esposito, S. Immune system and surgical site infection. *J. Chemother.* **13** (Spec 1), 12–16. 10.1179/joc.2001.13.Supplement-2.12 (2001). 10.1179/joc.2001.13.Supplement-2.12 11936355

[29] Qian, L. et al. The impact of body mass index on operative time in transoral endoscopic thyroidectomy vestibular approach for thyroid cancer. *Endocrine*. 10.1007/s12020-023-03616-z (2023). 38091199 10.1007/s12020-023-03616-z

[30] Opsomer, G. J., Vandeputte, F. J. & Sarac, C. Orthostatic retractor placement reduces operating time and post-operative inflammatory response during the learning curve of anterior approach THA. *J. Orthop.* **22**, 503–512. 10.1016/j.jor.2020.10.011 (2020). 33132623 10.1016/j.jor.2020.10.011 PMC7586062

[31] Herrmann, A. et al. Adhesions after laparoscopic myomectomy: incidence, risk factors, complications, and Prevention. *Gynecol. Minim. Invasive Ther.* **9**, 190–197. 10.4103/gmit.Gmit_87_20 (2020). 33312861 10.4103/GMIT.GMIT_87_20 PMC7713662

[32] Gallaway, K. E., Atadja, L. A. & Callan, A. K. Fever and systemic inflammatory response syndrome after wide resection of Pediatric Bone Sarcomas. *J. Pediatr. Orthop.* **42**, e783–e787. 10.1097/bpo.0000000000002181 (2022). 35552300 10.1097/BPO.0000000000002181

[33] Eckart, A. et al. Relationship of nutritional status, inflammation, and serum albumin levels during Acute illness: a prospective study. *Am. J. Med.* **133**, 713–722e717. 10.1016/j.amjmed.2019.10.031 (2020). 31751531 10.1016/j.amjmed.2019.10.031

[34] Yamada, S. et al. Very low protein diet enhances inflammation, malnutrition, and vascular calcification in uremic rats. *Life Sci.* **146**, 117–123. 10.1016/j.lfs.2015.12.050 (2016). 26764234 10.1016/j.lfs.2015.12.050

[35] Wiedermann, C. J. Hypoalbuminemia as Surrogate and Culprit of infections. *Int. J. Mol. Sci.* **22**10.3390/ijms22094496 (2021). 10.3390/ijms22094496 PMC8123513 33925831

